# Confinement Effects on Glass-Forming Aqueous Dimethyl Sulfoxide Solutions

**DOI:** 10.3390/molecules25184127

**Published:** 2020-09-09

**Authors:** Dominik Demuth, Melanie Reuhl, Moritz Hopfenmüller, Nail Karabas, Simon Schoner, Michael Vogel

**Affiliations:** Institute of Condensed Matter Physics, Technische Universität Darmstadt, 64289 Darmstadt, Germany; dominik.demuth@physik.tu-darmstadt.de (D.D.); melanie@nmr.physik.tu-darmstadt.de (M.R.); moritz.hopfenmueller@gmail.com (M.H.); nailkarabas@hotmail.com (N.K.); s-schoner@web.de (S.S.)

**Keywords:** confinement, aqueous solutions, glass transition, molecular dynamics, broadband dielectric spectroscopy, nuclear magnetic resonance

## Abstract

Combining broadband dielectric spectroscopy and nuclear magnetic resonance studies, we analyze the reorientation dynamics and the translational diffusion associated with the glassy slowdown of the eutectic aqueous dimethyl sulfoxide solution in nano-sized confinements, explicitly, in silica pores with different diameters and in ficoll and lysozyme matrices at different concentrations. We observe that both rotational and diffusive dynamics are slower and more heterogeneous in the confinements than in the bulk but the degree of these effects depends on the properties of the confinement and differs for the components of the solution. For the hard and the soft matrices, the slowdown and the heterogeneity become more prominent when the size of the confinement is reduced. In addition, the dynamics are more retarded for dimethyl sulfoxide than for water, implying specific guest-host interactions. Moreover, we find that the temperature dependence of the reorientation dynamics and of the translational diffusion differs in severe confinements, indicating a breakdown of the Stokes–Einstein–Debye relation. It is discussed to what extent these confinement effects can be rationalized in the framework of core-shell models, which assume bulk-like and slowed-down motions in central and interfacial confinement regions, respectively.

## 1. Introduction

The confinement of liquids to length scales of nanometers is very important in a wide array of technological fields, e.g., in nanotribology [1] and nanofluidics [2,3,4]. It is also found in biological settings like ion channels [5], membrane pores [6], and even inside cells owing to macromolecular crowding [7]. These examples illustrate that there is a multitude of confining matrices with numerous properties. A common distinction is between ‘hard’ [8,9,10] and ‘soft’ [11,12,13] confinements, depending on the potential mobility of their constituent particles [14,15]. Of particular interest are confined hydrogen-bonded liquids, which are omnipresent in applications and nature [16,17,18,19,20].

It is well established that nano-sized confinements strongly influence the structural and dynamical properties of liquids, including hydrogen-bonded compounds [21,22,23,24,25,26,27,28,29]. Prominent observations are the suppression of crystallization and changes in the glass transition temperatures $T_{g}$. Moreover, liquids in confinement tend to show enhanced dynamical heterogeneity. Explicitly, simulation studies found that molecular dynamics can be bulk-like in pore centers but orders of magnitude slower at pore walls [30,31,32]. Accordingly, experimental works employed core-shell models to describe motional inhomogeneity across confinements [33,34]. However, reorientation and diffusion dynamics may be affected to different degrees, as reports on a breakdown of the Stokes–Einstein–Debye relation for confined water indicate [35,36].

Further effects occur for hydrogen-bonded binary mixtures in confinement, which are especially relevant in various fields. They exhibit particularly complex structural and dynamical behaviors because each molecule may interact with molecules of the same and of the other kind as well as with the pore walls [37,38,39,40]. For example, the phase behavior can be affected by such multitude of interactions [41]. In particular, several studies found an increased tendency for microphase separation of confined binary mixtures as a consequence of preferential interactions with the pore walls [42,43,44,45]. Furthermore, it was reported that the dependence of the glass transition temperature $T_{g}$ on the composition differs for confined and bulk hydrogen-bonded mixtures [38].

Aqueous solutions of dimethyl sulfoxide (DMSO) already show intriguing properties in the bulk. Due to its amphiphilic nature, DMSO is a widely used aprotic solvent, which is readily miscible with polar and nonpolar compounds in wide ranges of compositions. Many properties of aqueous DMSO solutions do not depend monotonically on the water concentration, whereat the deviations from the ideal mixing behavior are most prominent for the eutectic composition with a 66mol% water fraction [46,47,48,49,50,51]. It was proposed that the nonideal mixing behavior originated in strong hydrogen bonds between DMSO oxygens and water hydrogens [51,52,53,54,55]. Moreover, it was found that aqueous DMSO solutions exhibited good glass-forming ability for a range of water concentrations around the eutectic composition, qualifying DMSO for use as a cryopreservation agent [56] and enabling dynamical studies in wide temperature ranges [57,58]. Thus, the high relevance paired with the nontrivial properties render aqueous DMSO solutions ideal model systems to ascertain confinement effects on hydrogen-bonded binary liquids.

Here, we investigate the influence of hard and soft confinements on the dynamics of aqueous DMSO solutions. Most studies are performed for the eutectic solution with 66mol% water so that its good glass-forming ability can be exploited to compare liquid dynamics in the bulk and confinement over broad temperature ranges. We employ the mesoporous silica MCM-41 and SBA-15, which feature defined cylindrical pores with adjustable diameters *d*, as hard confinements and we use the branched, hydrophilic polysaccharide ficoll and the small, globular protein lysozyme at sufficiently high concentrations as soft confinements. Ficoll, which proved to be useful to mimic macromolecular crowding in cells [27,59,60], is a spherical molecule with a size of ∼10 nm and a conformation intermediate between a solid sphere and a random coil [61,62]. Lysozyme is a single polypeptide of 129 amino acids, which has a molecular weight of 14.3 kDa and is folded in a compact, ellipsoidal structure with a long cleft. Both macromolecules show no overall motions but only internal motions at the studied compositions and, hence, they form disordered soft confinements. In the case of ficoll, we focus on the eutectic composition of the solution but study two samples with different solution:ficoll ratios, explicitly, with solution mass fractions of $w_{s}=40\;$wt% and $w_{s}=70\;$wt%, so as to change the typical distance between the macromolecules and, thus, the characteristic confinement size. For lysozyme confinement, we study the solution with 66mol% at a solvation level of $s=0.68\;g_{\mathrm{sol}}/g_{\mathrm{lys}}$ and we investigate a solution with 33mol% water at a comparable solvation level, see Section 4.1. This variation of the solvent composition allow us to realize potentially stabilizing or denaturating conditions for the protein and to check for possible effects on its dynamics. Unlike for the hard confinements, it is difficult to specify the sizes for the soft confinements due to the disordered arrangements and the irregular shapes of the involved macromolecules, but we expect that the solutions form very few solvation layers.

To ascertain the dynamical behaviors of the solutions on various time and length scales and in wide temperature ranges down to glassy arrest, we combine broadband dielectric spectroscopy (BDS) and nuclear magnetic resonance (NMR) studies. In the NMR experiments, the isotope selectivity of the method is exploited to single out the dynamical behaviors of the individual molecular species. Specifically, we perform ${}^{1}$H and ${}^{2}$H NMR measurements on solutions prepared from labeled compounds, explicitly, H${}_{2}$O-DMSO-$d_{6}$ and D${}_{2}$O-DMSO-$h_{6}$ mixtures. On the one hand, we combine spin-lattice relaxation (SLR) and stimulated echo experiment (STE) studies to investigate molecular rotational motion in a broad time window. On the other hand, we apply a static field gradient (SFG) to ascertain translational diffusion on micrometer scales. Thus, a comparison of the results allows us to obtain detailed insights into the relation of short-range and long-range dynamics, e.g., to test the validity of the Stokes–Einstein–Debye relation.

## 2. Results

### 2.1. Broadband Dielectric Spectroscopy

First, we employed BDS to gain insights into confinement effects on the dynamics of D${}_{2}$O-DMSO-$h_{6}$ mixtures. In doing so, we exploited that the complex dielectric permittivity $\varepsilon^{*}\left(\nu \right)=\varepsilon^{\prime }\left(\nu \right)-i\varepsilon^{\prime \prime }\left(\nu \right)$ informed us about the rotational correlation function

(1) $$F_{1}\left(t\right)\propto \langle P_{1}\left[\cos\theta \left(0\right)\right]P_{1}\left[\cos\theta \left(t\right)\right]\rangle$$

where $P_{1}$ denotes the first Legendre polynomial and the angle θ specifies the orientation of the molecular dipole moment [63,64].

**Figure 1:**
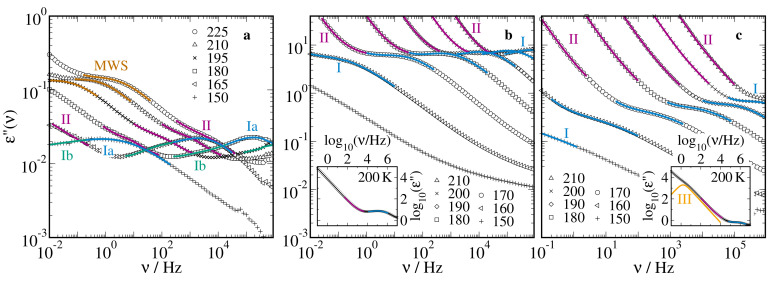
Dielectric loss spectra ε"(ν) of 66 mol% D${}_{2}$O-DMSO-$h_{6}$ solution in (**a**) silica pores with a diameter of d=2.8nm, (**b**) ficoll at a solution mass fraction of $w_{s}=70\;$wt%, and (**c**) lysozyme at a solvation level of $s=0.68\;g_{\mathrm{sol}}/g_{\mathrm{lys}}$. The temperatures are indicated in Kelvin. The colored lines and Roman numbers mark the individual relaxation processes. The insets show the dielectric loss spectra at 200 K in an extended frequency range.

**Figure 2:**
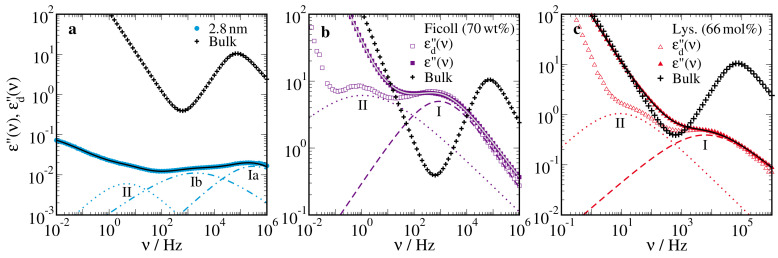
Dielectric loss spectra $\varepsilon^{\prime \prime }\left(\nu \right)$ of 66mol% D${}_{2}$O-DMSO-$h_{6}$ solution in various confinements at 180 K: (**a**) in silica pores with a diameter of d=2.8nm, (**b**) in ficoll at a solution mass fraction of $w_{s}=70\;$wt%, and (**c**) in lysozyme at a solvation level of $s=0.68\;g_{\mathrm{sol}}/g_{\mathrm{lys}}$. In each panel, the results for the bulk solution at 180 K are included [57]. The solid lines are fits of the data for the confined solutions to Equation (2). The dashed, dash-dotted, and dotted lines indicate the contributions of the individual Cole–Cole (CC) relaxation processes. In panels (**b**) and (**c**), the corresponding derivative of the real part of the dielectric permittivity, $-d\varepsilon^{\prime }\left(\nu \right)/d\ln\nu ∼\varepsilon_{d}^{\prime \prime }\left(\nu \right)$ is shown for comparison.

Figure 1 displays representative dielectric loss spectra $\varepsilon^{\prime \prime }\left(\nu \right)$ of 66mol% D${}_{2}$O-DMSO-$h_{6}$ in silica pores with a diameter of d=2.8nm, in ficoll at a solution fraction of $w_{s}=70\;$wt%, and in lysozyme at a solvation level of $s=0.68\;g_{\mathrm{sol}}/g_{\mathrm{lys}}$. In the silica confinement, we observed overall four BDS processes. A slow process with high intensity exited the experimental frequency window upon cooling at ∼195 K. In BDS studies of other confined hydrogen-bonded liquids [33,65], a process with similar properties was regarded as Maxwell–Wagner–Sillars (MWS) polarization. Following this assignment, we refrained from further analysis of this process and focused on the three faster processes. The two fastest ones, which we denoted as Ia and Ib for reasons discussed below, were close in position, yet resolvable upon closer inspection. In between the slowest and fastest processes, we observed a process II, which was partly covered by the MWS contribution. In line with previous BDS results for hydrated biological systems [66,67,68,69], the dielectric loss spectra of the solution in the ficoll and lysozyme confinements had a strong conductivity contribution, which is more evident in the insets. In both soft confinements, we found a single fast process, designated as process I, while the strong conductivity contribution interfered with a direct observation of slower relaxations. However, detailed analysis, e.g., consideration of the derivative of the real part, $-d\varepsilon^{\prime }\left(\nu \right)/d\ln\nu ∼\varepsilon_{d}^{\prime \prime }\left(\nu \right)$ [70], which was devoid of conductivity contributions, clearly unraveled the existence of a slower process II, see Figure 2. The BDS findings for the ficoll and lysozyme matrices differed in an even slower process III with a very high dielectric relaxation strength, which existed in the latter but not in the former confinement, see insets in Figure 1. A similar BDS process was reported in previous studies on various hydrated proteins [68,71,72], but its origin is still a matter of debate [73,74,75].

Further $\varepsilon^{\prime \prime }\left(\nu \right)$ data for other systems can be found in the Supplementary Materials. In detail, reducing the typical size of the confinements inside the ficoll matrix by decreasing the solvent concentration from $w_{s}=70\;$wt% to $w_{s}=40\;$wt%, we found a strong slowdown of molecular dynamics. Contrarily, changing the composition of the lysozyme-confined solution from 66mol% to 33mol% water, we observed no significant dependence of the dynamical behavior on the solvent composition. Below, we will discuss these effects in more detail based on correlation times obtained from a quantitative analysis of our BDS results.

In Figure 2, we compare the dielectric loss spectra $\varepsilon^{\prime \prime }\left(\nu \right)$ of confined and bulk 66mol% D${}_{2}$O-DMSO-$h_{6}$ solutions at 180 K. We see that the confined solutions showed more and broader relaxation processes than the bulk solution, implying that the related reorientation dynamics were more heterogeneous in the former than the latter samples. To quantify the observed differences with respect to the number, position, and shape of the relaxation processes, we fitted the complex dielectric permittivity of the confined solutions to a superposition of several Cole–Cole (CC) processes supplemented by a conductivity term:

(2) $$\varepsilon^{*}\left(\omega \right)=\varepsilon_{\infty }+\sum_{n}\frac{\Delta \varepsilon_{n}}{1+{\left(i\omega \tau_{\mathrm{cc},n}\right)}^{\alpha_{\mathrm{cc},n}}}-i\frac{\sigma_{\mathrm{dc}}}{\varepsilon_{0}\omega }\;.$$

Here, each CC relaxation process *n* is described by its relaxation strength $\Delta \varepsilon_{n}$, time constant $\tau_{\mathrm{cc},n}$, and width parameter $0<\alpha_{\mathrm{cc},n}\leq 1$. Moreover, $\sigma_{\mathrm{dc}}$ denotes the DC conductivity, $\varepsilon_{0}$ is the vacuum permittivity, and $\varepsilon_{\infty }$ is the high-frequency limit of the permittivity. This fit approach proved to be suitable in previous BDS studies of confined liquids [65,67,68]. Consistently, we see in Figure 2 that a superposition of CC processes well describes the dielectric loss spectra of the D${}_{2}$O-DMSO-$h_{6}$ solutions in the silica, ficoll, and lysozyme confinements. For the bulk solution, the temperature-dependent permittivity $\varepsilon^{*}\left(\nu \right)$ was analyzed in detail in previous work [57]. To enable straightforward comparison of the results for the confined and bulk solutions, we considered peak correlation times $\tau_{n}$ throughout this contribution. For the confined solutions, $\tau_{n}$ can be directly identified with the time constant of the CC function, $\tau_{\mathrm{cc},n}$. For the bulk solution, we determined the peak correlation times from the results of the previous study [57], where the mean correlation times were given.

**Figure 3:**
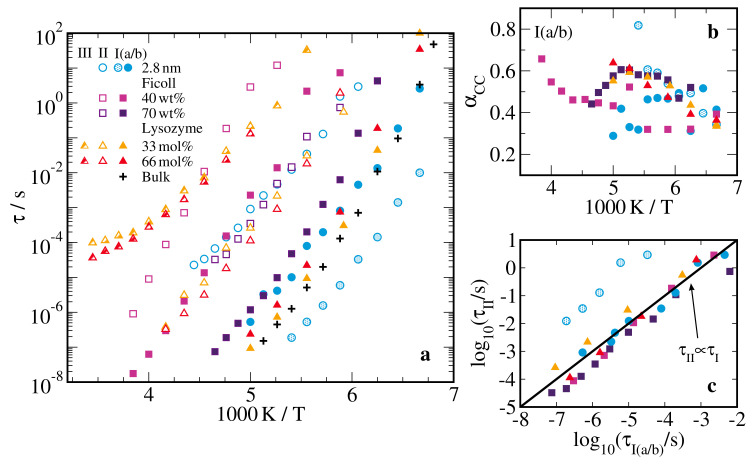
Results from broadband dielectric spectroscopy (BDS) studies on 66 mol% D${}_{2}$O-DMSO-$h_{6}$ solution in various confinements and in the bulk [57]: (**a**) correlation times $\tau_{n}$ of all observed relaxation processes and (**b**) width parameters $\alpha_{\mathrm{cc},n}$ of processes I and Ia/Ib. Panel (**c**) shows $\tau_{\mathrm{II}}$ as a function of $\tau_{I}$ or $\tau_{\mathrm{Ia}/\mathrm{Ib}}$. The solid line indicates a linear dependence. The used confinements are silica pores with a diameter of d=2.8 nm, ficoll at solution mass fractions of $w_{s}=40\;$wt% and $w_{s}=70\;$wt%, and lysozyme at a solvation level of $s=0.68\;g_{\mathrm{sol}}/g_{\mathrm{lys}}$. For the lysozyme confinement, results for 33 mol% D${}_{2}$O–DMSO-$h_{6}$ solution are included for comparison.

In Figure 3a, we present the resulting temperature-dependent BDS peak correlation times $\tau_{n}$ of all studied confined and bulk D${}_{2}$O-DMSO-$h_{6}$ solutions. We see that the correlation times of the processes Ia/b in the silica confinement and of process I in the soft confinements were similar to those of the bulk process, which characterized the α relaxation of the bulk solution and vitrified at the glass transition temperature of $T_{g}=146$ K [57]. These similarities imply that the former processes, which we will abbreviate as I(a/b) processes in the following if a distinction is not necessary, were related to the structural relaxation of the confined liquids. Yet, the I(a/b) processes of the confined solutions and the α process of the bulk solution could differ with respect to the degree of deviations from Arrhenius behavior. For the silica pores, we observed that $\tau_{\mathrm{Ia}}$ was shorter than the bulk $\tau_{\alpha }$, while $\tau_{\mathrm{Ib}}$ was longer except for temperatures near the glassy arrest. Both soft confinements caused a slowdown of the solution dynamics at all studied temperatures, but the effect was stronger for ficoll than for lysozyme. However, $\tau_{I}$ in the ficoll matrix strongly depended on the concentration of the solution; explicitly, it increased by about two orders of magnitude when $w_{s}$ decreased from 70 wt% to 40 wt% and, hence, the solution became confined to narrow interfacial layers. On the other hand, in the lysozyme matrix, $\tau_{I}$ decreased only moderately, when we reduced the water content of the solution from 66 mol% to 33 mol%.

In addition, our fit approach allowed us to quantify the shape of the relaxation processes of the confined solutions. In Figure 3b, we see that the I(a/b) processes are characterized by small width parameters in the range $\alpha_{\mathrm{cc},}\;I(a/b)\approx$ 0.3–0.6, indicating that the related rotational motions occurred on broadly distributed time scales. Consistently, previous studies on hydrogen-bonded liquids in various types of confinements reported broad distributions of correlation times [14,15,28]. More precisely, the relaxation processes of confined liquids showed a broadening on the low-frequency flank, which did not occur for the α processes of bulk liquids. This broadening was mainly caused by slow molecules near the confining walls [32,44,76], as was anticipated in core-shell models and confirmed in systematic filling-level and solvation-level dependent studies on neat confined liquids [65,77,78,79]. Here, the CC distribution is used to phenomenologically consider this effect for the confined solutions, while the Cole-Davidson distribution proved to be useful to describe the α process of glass-forming bulk liquids.

The slower processes of the confined solutions do not have counterparts in the bulk. Process II exists in all studied confinements. However, the ratio of the dielectric relaxation strengths $\Delta \varepsilon_{\mathrm{II}}/\Delta \varepsilon_{I(a/b)}$ varies between the systems. Specifically, process II strongly grows with respect to processes I(a/b) when moving from silica over ficoll to lysozyme confinement, see Figure 2. For all matrices, process II is roughly 3–4 orders of magnitude slower than processes I(a/b). Plotting $\tau_{\mathrm{II}}$ as a function of $\tau_{I(a/b)}$, we observe in Figure 3c that these processes have a similar temperature dependence. This finding suggests that process II is related to the structural reorganization inside the confinements in one way or another but, owing to the intricate interpretation of the dielectric permittivity of inhomogeneous media, its microscopic nature remains elusive [80,81]. Therefore, we refrain from a more detailed analysis. Process III is singular to the lysozyme mixtures, in accordance with the conjecture that it is related to solvation-enabled protein dynamics [73,74,75]. It has a weaker temperature dependence than the faster processes. However, it hardly changes when the composition of the solution is varied from 66 mol% to 33 mol% water and, hence, we do not find evidence that lysozyme dynamics is substantially different under stabilizing or denaturating conditions.

### 2.2. ${}^{2}$H NMR

Next, we performed ${}^{2}$H NMR experiments to investigate reorientation dynamics in 66 mol% D${}_{2}$O-DMSO-$h_{6}$ solutions. Owing to the lack of deuterons, DMSO-$h_{6}$ did not contribute to these measurements. Therefore, our ${}^{2}$H NMR approaches to silica confinements selectively observed D${}_{2}$O dynamics. However, for the soft confinements, we needed to consider that ficoll and lysozyme have exchangeable protons in O–H and N–H bonds, respectively. We expected these groups to receive deuterons from D${}_{2}$O via chemical exchange and, hence, to contribute to the measured signals. Thus, our ${}^{2}$H NMR experiments probed the quadrupolar frequencies $\omega_{Q}$ of deuterons in water O–D bonds and, for soft confinements, in ficoll O–D bonds or lysozyme N–D bonds, which are approximately given by [82]

(3) $$\omega_{Q}=\pm \frac{\delta }{2}\left(3\cos^{2}\theta -1\right)\propto P_{2}\left(\cos\theta \right)\;.$$

Here, the angle θ describes the orientation of the O–D or N–D bond relative to the applied external magnetic field $B_{0}$, and δ characterizes the strength of the respective quadrupolar interaction. Since the quadrupolar frequency is proportional to the second Legendre polynomial $P_{2}\left(\cos\theta \right)$, fluctuations of $\omega_{Q}$ provide access to the correlation function

(4) $$F_{2}\left(t\right)\propto \langle P_{2}\left[\cos\theta \left(0\right)\right]P_{2}\left[\cos\theta \left(t\right)\right]\rangle \;.$$

We note that the 66 mol% D${}_{2}$O-DMSO-$h_{6}$ bulk solution was characterized in previous ${}^{2}$H NMR studies [57]. Here, we perform additional measurements to cover the temperature range in greater detail but use the same evaluation methods. Therefore, we refrain from detailed description of these measurements and analyses. However, the experimental data and the resulting correlation times are included in the respective graphs.

#### 2.2.1. ${}^{2}$H Spin-Lattice Relaxation

${}^{2}$H SLR is particularly sensitive to molecular reorientation in the nanosecond regime [83]. In these experiments, we observed the ${}^{2}$H magnetization buildup M(t). Considering that different deuteron species *n* can show different SLR behaviors, we fitted the experimental data using one or, if required, a sum of two stretched exponential functions

(5) $$M\left(t\right)=M_{\infty }-\sum_{n}M_{n}\exp\left[-{\left(t/T_{1,n}\right)}^{\beta_{n}}\right]\;.$$

Here, each SLR step is characterized by the SLR time $T_{1,n}$, the stretching parameter $\beta_{n}$, and its share $M_{n}$ on the equilibrium magnetization $M_{\infty }$. From the fit results, we calculated the mean SLR times $\langle T_{1,n}\rangle =T_{1,n}/\beta_{n}\cdot \Gamma \left(1/\beta_{n}\right)$ to account for possible nonexponentiality.

**Figure 4:**
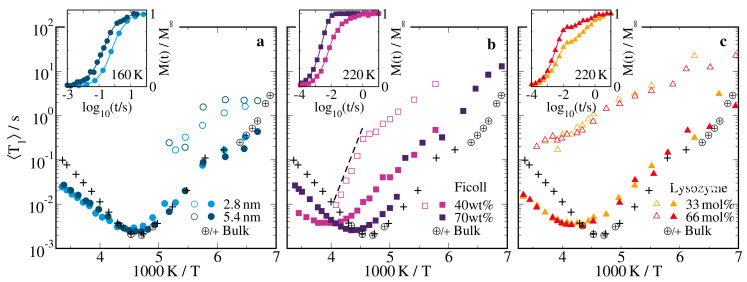
Temperature-dependent mean ${}^{2}$H SLR times $\langle T_{1,n}\rangle$ of 66 mol% D${}_{2}$O-DMSO-$h_{6}$ solution in (**a**) silica pores with diameters of d=2.8 nm and d=5.4 nm, (**b**) ficoll at solution mass fractions of $w_{s}\;=\;70\;$wt% and $w_{s}=40\;$wt%, and (**c**) lysozyme at a solvation level of $s=0.68\;g_{\mathrm{sol}}/g_{\mathrm{lys}}$. For the lysozyme matrix, results for a solution with 33 mol% water are also included. In all panels, data for the 66 mol% D${}_{2}$O-DMSO-$h_{6}$ bulk solution are shown for comparison, where present and previous [57] results are marked by crosses and circled crosses, respectively. The insets show exemplary normalized buildup curves $M\left(t\right)/M_{\infty }$. In panel (**b**), the dashed line indicates an Arrhenius law with an activation energy of $E_{a}=65\;\mathrm{kJ}/\mathrm{mol}$. All measurements were performed at the Larmor frequency $\omega_{0}=2\pi \cdot 46.1\;$MHz.

**Figure 5:**
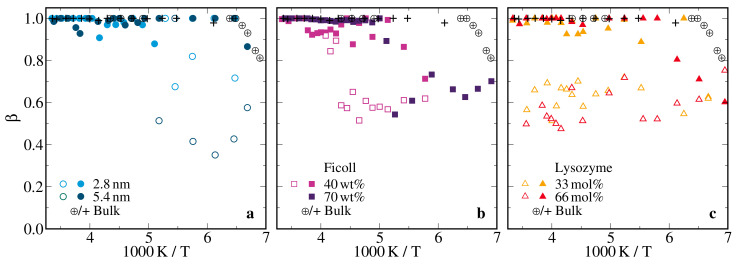
Temperature-dependent stretching parameters $\beta_{n}$ obtained from ${}^{2}$H SLR studies on 66 mol% D${}_{2}$O-DMSO-$h_{6}$ solution in (**a**) silica pores with diameters of d=2.8 nm and d=5.4 nm, (**b**) ficoll at solution mass fractions of $w_{s}=70\;$wt% and $w_{s}=40\;$wt%, and (**c**) lysozyme at a solvation level of $s=0.68\;g_{\mathrm{sol}}/g_{\mathrm{lys}}$. For the lysozyme matrix, results for a solution with 33 mol% water are also included. In all panels, data for the 66 mol% D${}_{2}$O-DMSO-$h_{6}$ bulk solution are shown for comparison, where present and previous [57] results are marked by crosses and circled crosses, respectively.

Figure 4 and Figure 5 compile the results of this SLR analysis for all studied samples. At sufficiently high temperatures, we observed a single exponential SLR, i.e., the index *n* could be dropped, β=1, and $\langle T_{1}\rangle \equiv T_{1}$. Upon cooling, bimodal SLR developed. While the faster (f) SLR step continued the high-temperature behavior, the slower (s) SLR step was smaller in most cases and set in as a separate phenomenon. Explicitly, for all confinements, $T_{1,f}$ was a continuation of $T_{1}\left(T\right)$ and $\beta_{1,f}=1$ was found except for low temperatures. The latter finding is typical of liquid-like solution dynamics, which is sufficiently fast to average possibly distinct dynamical behaviors in different regions of the confinements on the $T_{1,f}$ time scale. By contrast, $T_{1,s}$ was much longer and $\beta_{1,s}\approx 0.6$ at all temperatures. The latter result indicates nonergodic behavior on the time scale of $T_{1,s}$ and, hence, solid-like components. For all confined and bulk solutions, we found clear $T_{1}$ or $T_{1,f}$ minima, indicating that the correlation time τ of the probed reorientation dynamics was of the order of the inverse Larmor frequency $\omega_{0}$ and hence, in the nanoseconds regime. For a determination of temperature-dependent correlation times from the ${}^{2}$H SLR results, we refer to Section 2.3.

In Figure 4a, we compare the ${}^{2}$H SLR times for the solutions in silica pores with diameters of 2.8 nm and 5.4 nm with the bulk behavior. In both hard confinements, we observed exponential SLR described by a single $T_{1}$ value above 190 K. When the pore diameter was reduced, the position of the $T_{1}$ minimum mildly shifted to higher temperatures, revealing a moderate slowdown of the average reorientation dynamics, and the height of the $T_{1}$ minimum weakly grew, indicating that the heterogeneity of this motion increased [83]. While $T_{1}$ was significantly shorter for the confined solutions than the bulk solution at temperatures above the minimum, there was hardly any difference below. To rationalize these observations, we need to consider that the slow and fast parts of distributions G(logτ) govern the ${}^{2}$H SLR behaviors at high and low temperatures, respectively. Thus, the disparate $T_{1}$ values above the minimum and the similar values below imply that the long-time parts of the distributions G(logτ) differ for the confined and bulk solutions, while the short-time parts resemble each other. Thus, consistent with the outcome of our BDS studies, the SLR findings suggested that the D${}_{2}$O reorientation dynamics of the confined solutions was slowed down near the pore walls, leading to significant broadening of the low-frequency flank of $\varepsilon^{\prime \prime }\left(\nu \right)$ and of the long-time flank of G(logτ), while it weakly deviated from that of the bulk solution in the pore center. Below 190 K, we found bimodal SLR behavior, which was essentially independent of the pore diameter. A similar bimodality was reported for neat hydrogen-bonded liquids in silica pores at low temperatures [84,85,86,87,88]. However, despite detailed analyses, the origin is still unclear. It was argued that the fast and slow SLR steps result from, respectively, liquid and solid phases which coexisted in confinement as a consequence of partial crystallization. Alternatively, it was proposed to rationalize the bimodality in the framework of core-shell models. Thus, it remains an open question whether or not the $T_{1,f}$ and $T_{1,s}$ steps are related to the Ia and Ib BDS processes in the silica confinement, which, in turn, may characterize the dynamics in central and interfacial pore regions.

In Figure 4b,c, we see that the ficoll and lysozyme confinements had larger effects on ${}^{2}$H SLR. Specifically, the shifts in the position and the height of the $T_{1}$ minimum were stronger in these soft confinements than in the silica pores, indicating that the slowdown and the heterogeneity of the solution were further enhanced. These effects were particularly prominent when we decreased the solution fraction $w_{s}$ in the ficoll matrix from 70 to 40 wt% and, thus, reduced the confinement size. For a more precise discussion of the ${}^{2}$H SLR results for the soft confinements, it is, however, necessary to consider that water and matrix deuterons will produce fast and slow SLR steps if the chemical exchange between these species is slow on the time scale of the magnetization buildup. Thus, despite the observation of an unpartitioned BDS process I, SLR could be bimodal in soft confinements. However, the bimodality, if observed, did not result from dynamically distinguishable solution fractions but rather from the chemically different deuteron species.

In the lysozyme confinement, two steps could be clearly resolved in the whole temperature range and, hence, the associated $T_{1,f}$ and $T_{1,s}$ times could be interpreted in term of water and lysozyme, respectively. We observed that neither of these SLR times changes significantly, when the composition of the solution was altered. Thus, the $T_{1,f}$ results showed that the reorientation dynamics of D${}_{2}$O was slower in the confined solutions than in the bulk solution and that it was hardly affected when the water content in the solution was reduced from 66 to 33 mol%, in agreement with the results for BDS process I. Likewise, the $T_{1,s}$ data indicated that the rotational motion of the N–D lysozyme bonds was not affected by this change in the composition of the solution, consistent with the findings for BDS process III. In accordance with ${}^{2}$H SLR findings for other proteins [89,90,91], $T_{1,s}$ was long and continuously increased upon cooling, indicating that lysozyme did not show significant mobility in the nanoseconds regime but only restricted motion such as caged dynamics so that the observed independence of the solvent composition was plausible.

In the ficoll confinement, chemical exchange strongly affected the ${}^{2}$H SLR behavior. For the solution fraction $w_{s}=70\;$wt%, monomodal SLR was observed at all studied temperatures, indicating that chemical exchange was sufficiently fast to establish a common SLR time of water and ficoll deuterons. Hence, the $T_{1}$ minimum could not be attributed to a particular component but reflected an average behavior of both deuteron species. For $w_{s}=40\;$wt%, bimodal SLR set in near 250 K. While $T_{1,f}$ increased uniformly upon cooling below this temperature, $T_{1,s}$ showed a kink at ∼220 K. The latter finding suggested that the time scale of the chemical exchange crossed that of $T_{1,s}$ at this temperature. Consistently, the temperature dependence of $T_{1,s}$ above 220 K was characterized by an activation energy of 65 kJ/mol, which was previously reported for chemical exchange in sucrose solutions [92]. Thus, chemical exchange interfered with straightforward interpretation of the SLR times in terms of molecular dynamics not only for $w_{s}=70\;$wt% but also for $w_{s}=40\;$wt% at least in the minimum region.

#### 2.2.2. ${}^{2}$H Solid-Echo Intensities

**Figure 6:**
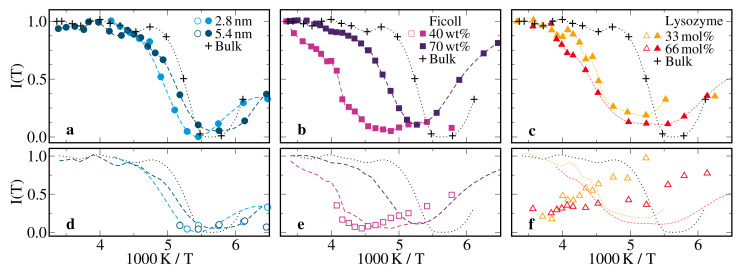
Temperature dependence of the normalized ${}^{2}$H NMR solid-echo intensities $I_{n}\left(T\right)$ for 66 mol% D${}_{2}$O-DMSO-$h_{6}$ solution in (**a**,**d**) silica pores with diameters of d=2.8 nm and d=5.4 nm, (**b**,**e**) ficoll at solution mass fractions of $w_{s}=70\;$wt% and $w_{s}=40\;$wt%, and (**c**,**f**) lysozyme at a solvation level of $s=0.68\;g_{\mathrm{sol}}/g_{\mathrm{lys}}$, where results for solutions with 66 mol% and 33 mol% water are compared. The intensities were obtained from the amplitudes of the ${}^{2}$H SLR steps and corrected for the Curie factor, explicitly, $I_{n}\left(T\right)=M_{n}\left(T\right)\cdot T$. The upper and lower panels show the results for the faster and slower SLR steps, respectively. The corresponding data for the 66 mol% D${}_{2}$O-DMSO-$h_{6}$ bulk solution are included for comparison. Identical lines are shown in the upper and lower panes as guides for the eye.

Further information about the reorientation dynamics of the bulk and confined D${}_{2}$O-DMSO-$h_{6}$ solutions can be gained from the temperature dependence of the ${}^{2}$H solid-echo intensity (SEI) I(T) [93,94]. This approach exploits that the applied solid-echo pulse sequence looses performance when molecular dynamics during the dephasing and rephasing periods of the measurement occur. Specifically, the SEI is minimal for correlation times τ≈1μs [89]. Here, we considered that dynamically distinguishable deuteron species could exist and determined separate intensities $I_{f}\left(T\right)$ and $I_{s}\left(T\right)$ when fast and slow ${}^{2}$H SLR steps could be resolved. The results are compiled in Figure 6. We see clear $I_{f}\left(T\right)$ minima, which indicate that D${}_{2}$O reorientation crossed the microseconds regime. The minima changed only mildly in position and height when confining the solution to the silica pores, while the shifts were stronger in the soft confinements. Thus, the SEI analysis confirmed the above BDS and SLR results for the confinement effects on the solution dynamics. More detailed comparisons will be performed in Section 2.3. By contrast, $I_{s}\left(T\right)$ increased weakly and continuously upon cooling, with the possible exception of the ficoll confinement. Different explanations can be given to rationalize this increase. It may indicate that the related molecular dynamics was too slow to produce an intensity minimum in the temperature range, where distinguishable SLR behaviors existed. Alternatively, it may imply that increasing fractions of the confined solutions showed solid-like dynamics, e.g., due to adsorption at the silica or macromolecular surfaces.

#### 2.2.3. ${}^{2}$H Stimulated-Echo Experiments

Next, we performed ${}^{2}$H STE studies to investigate slow D${}_{2}$O reorientation on time scales of ∼10${}^{-4}$–10${}^{0}\;$s. In these measurements, we directly correlated the quadrupolar frequencies $\omega_{Q}$ and, thus, the molecular orientations θ during two short evolution times $t_{e}$, which were separated by a variable mixing time $t_{m}$. Specifically, we measured the rotational correlation function [82,83]

(6) $$F_{2}^{\mathrm{ss}}\left(t_{m}\right)\propto \langle \sin\left[\omega_{Q}\left(0\right)t_{e}\right]\sin\left[\omega_{Q}\left(t_{m}\right)t_{e}\right]\rangle \;.$$

In doing so, we used short evolution times $t_{e}\to 0$ so that the STE decays yielded $F_{2}^{\mathrm{ss}}\left(t_{m}\right)\propto F_{2}\left(t_{m}\right)$, see Equation (3).

**Figure 7:**
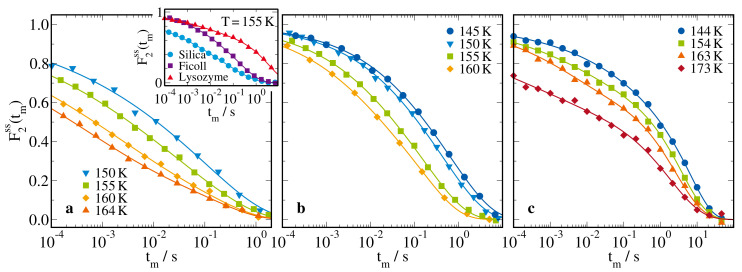
Correlation functions $F_{2}^{\mathrm{ss}}\left(t_{m}\right)$ of 66 mol% D${}_{2}$O-DMSO-$h_{6}$ solution in (**a**) in silica pores with a diameter of d=2.8 nm, (**b**) ficoll at a solution mass fractions of $w_{s}=70\;$wt%, and (**c**) lysozyme at a solvation level of $s=0.68\;g_{\mathrm{sol}}/g_{\mathrm{lys}}$. In all measurements, the evolution times were set to $t_{e}=4$–5μs to achieve $F_{2}^{\mathrm{ss}}\left(t_{m}\right)\propto F_{2}\left(t_{m}\right)$. The lines are fits to Equation (7). The inset compares $F_{2}^{\mathrm{ss}}\left(t_{m}\right)$ of the studied samples at T=155K.

Figure 7 shows ${}^{2}$H STE results for the 66 mol% D${}_{2}$O-DMSO-$h_{6}$ solution in the different types of confinements. For all samples, $F_{2}^{\mathrm{ss}}\left(t_{m}\right)$ shifted to longer times upon cooling. Moreover, the correlation functions were strongly stretched, confirming the high dynamical heterogeneity of the confined solutions. However, we did not observe bimodal decays, which may be expected based on our BDS and SLR findings but may remain unresolved due to the strong broadening of the relaxation processes in combination with the limited width of the experimental time window. For a quantitative analysis, we considered that, in addition to molecular reorientation, spin relaxation could lead to STE decays and fit the experimental data to

(7) $$F_{2}^{\mathrm{ss}}\left(t_{m}\right)=\left\{F_{\infty }+\left(1-F_{\infty }\right)\exp\left[-{\left(t_{m}/\tau_{K}\right)}^{\beta_{K}}\right]\right\}\exp\left[-{\left(t_{m}/T_{1Q}\right)}^{\beta_{Q}}\right]\;.$$

Here, we assume that the decay owing to molecular reorientation, the first factor can be described by a Kohlrausch function with time constant $\tau_{K}$ and stretching parameter $\beta_{K}$. Moreover, we allow for a residual correlation $F_{\infty }$ to account for possible anisotropic motions or immobile components. Finally, we consider that the damping due to spin relaxation, the second factor, involved the decay of the alignment state existing during the mixing time $t_{m}$ of this experiment [83], which is described by the parameters $T_{1Q}$ and $\beta_{Q}$. In Figure 7, we see that the experimental data were well described by this fit approach. The resulting correlation times will be presented in the next section. The obtained small stretching parameters confirmed the prominent nonexponentiality of the decays, explicitly, $\beta_{K}\approx 0.23$ in silica confinement and $\beta_{K}\approx 0.30$–0.35 in ficoll and lysozyme matrices. For the soft confinements, it was necessary to consider that ficoll and lysozyme contained deuterons but did not show reorientation dynamics on the STE time scale at the studied temperatures. Therefore, we expected that these matrix deuterons resulted in enhanced residual correlations. Accordingly, we found $F_{\infty }\approx 0.5$ for the lysozyme confinement. By contrast, we did not observe increased $F_{\infty }$ values for the ficoll matrix. This difference is also evident from a direct comparison of the $F_{2}^{\mathrm{ss}}\left(t_{m}\right)$ decays at 155 K in the inset. We speculate that the discrepancy relating to the residual correlation resulted because of faster chemical exchange for the ficoll than the lysozyme deuterons.

### 2.3. Rotational Correlation Times

To finish our studies on the molecular rotational dynamics of the confined solutions, we determined peak correlation times τ of D${}_{2}$O reorientation in a broad range from ambient temperatures down to the glassy arrest from our ${}^{2}$H NMR data, which can be compared with the results for the BDS processes I(a/b).

For quantitative ${}^{2}$H SLR analysis, we used the fact that, in the case of exponential magnetization buildup, $T_{1}$ is related to molecular reorientation via [95]

(8) $$\frac{1}{T_{1}}=\frac{2}{15}\delta^{2}\left[J_{2}\left(\omega_{0}\right)+J_{2}\left(2\omega_{0}\right)\right]$$

where $J_{2}\left(\omega \right)$ is the spectral density, i.e., the Fourier transform of the correlation function $F_{2}\left(t\right)$. Moreover, we exploited the knowledge about the strength of the quadrupolar coupling for D${}_{2}$O, δ=2π·161 kHz, from previous ${}^{2}$H NMR line-shape analysis [84,85,86,87] and assumed that $J_{2}\left(\omega \right)$ has Cole–Cole form, as is motivated by the above BDS results, so that the width parameter $\alpha_{\mathrm{cc}}$ could be determined from the height of the $T_{1}$ minimum [83]. We obtained $\alpha_{\mathrm{cc}}=0.62$ and $\alpha_{\mathrm{cc}}=0.69$ for the silica pores with d=2.8nm and d=5.4 nm, respectively, $\alpha_{\mathrm{cc}}=0.66$ for the ficoll matrix at $w_{s}=70\;$wt%, and $\alpha_{\mathrm{cc}}\approx 0.53$ for both the ficoll matrix at $w_{s}=40\;$wt% and the lysozyme confinement. In accordance with the above BDS results, these small width parameters indicate prominent dynamical heterogeneity, in particular, for narrow soft confinements. Using the thus determined spectral densities $J_{2}\left(\omega \right)$ in Equation (8), the measured $T_{1}$ values could be directly translated into the peak correlation times $\tau \equiv \tau_{\mathrm{cc}}$. We restricted this analysis to temperatures in the vicinity of the $T_{1}$ minimum, where the resulting correlation times hardly depended on the exact shape of the spectral density. To obtain synonymous time constants from the ${}^{2}$H STE study, we used the $\tau_{K}$ and $\beta_{K}$ parameters obtained from the Kohlrausch fit to calculate peak correlation times according to [28]

(9) $$\tau /\tau_{K}=1.785-0.871\beta_{K}-0.029\beta_{K}^{2}+0.114\beta_{K}^{3}\;.$$

**Figure 8:**
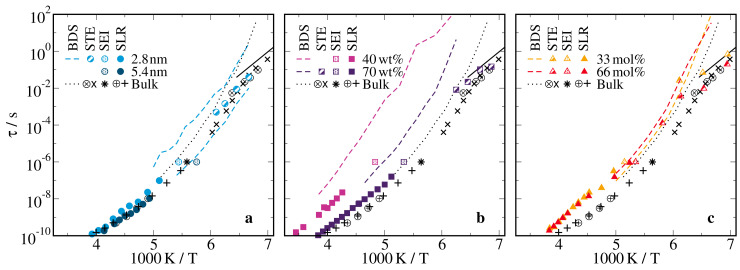
Temperature-dependent correlation times $\tau_{n}$ obtained from BDS and ${}^{2}$H NMR studies on 66 mol% D${}_{2}$O-DMSO-$h_{6}$ solution in (**a**) silica pores with diameters of d=2.8 nm and d=5.4 nm, (**b**) ficoll at solution mass fractions of $w_{s}=70\;$wt% and $w_{s}=40\;$wt%, and (**c**) lysozyme at a solvation level of $s=0.68\;g_{\mathrm{sol}}/g_{\mathrm{lys}}$. For the lysozyme matrix, results for a solution with 33 mol% water are also shown. For clarity, the displayed BDS results are limited to the I(a/b) processes. In all panels, data for the 66 mol% D${}_{2}$O-DMSO-$h_{6}$ bulk solution are included for comparison, where previous [57] results are shown as circled symbols. The solid lines indicate an Arrhenius temperature dependence with the activation energy $E_{a}=0.5$ eV.

In Figure 8, we display the BDS results for process I(a/b) and the bulk α process together with the ${}^{2}$H SLR, SEI, and STE correlation times of the confined and bulk solutions. The dynamics of the 66 mol% D${}_{2}$O-DMSO-$h_{6}$ bulk solution were ascertained in more detail in previous BDS and ${}^{2}$H NMR studies [57]. It was found that the α process showed the characteristic Vogel–Fulcher–Tammann (VFT) temperature dependence of many molecular glass-forming liquids. In the weakly supercooled regime, the BDS and ${}^{2}$H NMR results for $\tau_{\alpha }$ differed by a factor of ∼3, which can be rationalized when assuming isotropic rotational diffusion as mechanism for the motion and considering that these methods probed the rotational correlation functions $F_{1}$ and $F_{2}$, respectively. Upon approaching $T_{g}$, a faster β process was reported to decouple from the α process [57]. It was argued that the former may be the inherent Johari–Goldstein β process of glass-forming liquids or the ν process of water in aqueous systems, which shows an Arrhenius temperature dependence with the common activation energy of ∼0.5 eV and is, thus, often denoted as universal water relaxation [14,15,96]. These findings were confirmed by the additional ${}^{2}$H NMR data obtained for the bulk solution in the present contribution.

In the silica and lysozyme confinements, BDS and ${}^{2}$H NMR yielded consistent results for the reorientation dynamics of the D${}_{2}$O-DMSO-$h_{6}$ solution. In the weakly supercooled regime, the ${}^{2}$H SLR correlation times indicated that the solution dynamics, or more precisely, the D${}_{2}$O reorientation, in the wider silica pores resembled that in the bulk, while there was a mild slowdown in the narrower silica pores and a stronger one in the lysozyme matrix. At room temperature, the latter effects apparently diminished but we expect that this was an artifact owing to some uncertainties relating to the exact shape of the spectral density $J_{2}\left(\omega \right)$, which became relevant at temperatures farther away from the $T_{1}$ minimum at ∼220 K. Our findings for the confinement-dependent slowdown were confirmed for the D${}_{2}$O reorientation on the microseconds time scale by the results of the ${}^{2}$H SEI analysis. For the lysozyme confinement, the BDS correlation times continued the ${}^{2}$H SLR and SEI data, i.e., there was no evidence for the difference by the factor of ∼3 observed for the bulk solution. This suggests that the reorientation mechanism differed for the confined and bulk solutions, specifically, that large-angle jumps prevailed in the confinements so that differences between $F_{1}$ and $F_{2}$ correlation functions vanished, while small-angle jumps dominated in the bulk, including the limiting case of diffusive rotational motion. Consistently, large-angle jump mechanisms were reported for neat water and glycerol in silica and protein confinements [79,84,85,89]. For the silica pores, a detailed comparison of BDS and ${}^{2}$H SLR and SEI results was hampered by the fact that the Ia and Ib processes were not resolved in the NMR approaches.

In the deeply supercooled regime, the dynamical behaviors inside the confinements were more complex because the bulk solution showed α and β processes, which were barely resolved, and the confined solutions exhibited coexisting Ia and Ib processes in the silica pores, which were not distinguishable in the lysozyme and ficoll matrices. Thus, the ${}^{2}$H STE decays of the confined solutions could be affected by both α and β processes. Accordingly, we found that the ${}^{2}$H STE correlation times lay in between $\tau_{\alpha }$ and $\tau_{\beta }$ of the bulk solution, with a tendency to agree with the former and latter at higher and lower temperatures of the highly viscous regime. Moreover, they did not follow the VFT behavior of the BDS data but showed Arrhenius-like behavior, indicating that the D${}_{2}$O reorientation probed by ${}^{2}$H NMR started to decouple from the α process, as was reported for the bulk solution [57]. The temperature dependence were roughly consistent with the universal activation energy of ∼0.5 eV for the ν process of water, corroborating this conjecture. Likewise, the ${}^{2}$H STE analysis did not resolve the Ia and Ib processes and, hence, it did not provide insights into the origin of these BDS phenomena. Two relaxation processes with similar properties were observed for neat hydrogen-bonded liquids in silica confinement [33,86,88,97,98]. While they were interpreted in terms of core-shell models for confined alcohols [33,97], they were attributed to coexisting liquid and solid phases or high-density and low-density liquid phases for confined water [78,86,98]. In our case of binary mixtures, another possible explanation resulted from the finding that preferential interactions with the pore walls could lead to microphase separation and, thus, to bimodal dynamics [28,29,40,43,44,99,100].

For both ficoll mixtures, the BDS and ${}^{2}$H SLR and SEI correlation times differed by more than an order of magnitude. This large discrepancy could not be reconciled solely by the fact that different correlation functions were probed and, hence, it points to prominent effects of chemical exchange on the observations, consistent with the above discussion relating to the magnetization buildup. The relevance of these effects was expected to differ in BDS and ${}^{2}$H NMR and can depend on the dipole moments and deuteron fractions of the components. Either way, the observation that the correlation times of the α process were much longer in the ficoll confinements than in the bulk was, at least in part, caused by the fact that chemical exchange averaged the dynamical behaviors of the solvent and ficoll. Contrarily, in the ${}^{2}$H STE studies, we observed hardly any confinement effects for the β process, supporting the conjecture of its universal nature.

### 2.4. Self-Diffusion Coefficients

Next, we performed ^1^H SFG NMR measurements to investigate the translational diffusion in the confined and bulk solutions. For this purpose, we applied a magnetic field with a static gradient *g* along the *z* axis, $B\left(z\right)=B_{0}+gz$, so that the ${}^{1}$H Larmor frequency depended on the nuclear position according to ω(z)=γB(z), where γ is the gyromagnetic ratio. Under these circumstances, self-diffusion resulted in frequency changes, which could be observed in STE studies. Explicitly, for free diffusion of the bulk solution, the SFG STE decays depended on the self-diffusion coefficient *D* according to [101,102]

(10) $$S\left(t_{m},t_{e}\right)\propto \exp\left(-q^{2}Dt_{d}\right)$$

where the effective diffusion time amounted to $t_{d}=t_{m}+2t_{e}/3$ and the generalized scattering vector $q=\gamma gt_{e}$ determined the length scale of the diffusion measurements. The high field gradients *g* available from our specially designed setup allowed us to adjust the experimental length scale in the range ∼ 0.1–10 μm [103]. Moreover, in our ${}^{1}$H SFG STE approach, it was possible to investigate the components of the solutions separately by exploiting the isotope selectivity and using labeled compounds H${}_{2}$O-DMSO-$d_{6}$ and D${}_{2}$O-DMSO-$h_{6}$. Unlike in the bulk, the diffusion in the silica pores was restricted by impenetrable walls. Specifically, the length scale of the diffusion measurements was larger than the pore diameters, but smaller than the pore lengths [36]. In such situations, NMR diffusometry merely probed molecular displacements along the axes of the cylindrical pores, i.e., we observed one-dimensional diffusion, leading to STE decays [102,104]

(11) $$S\left(t_{m},t_{e}\right)\propto \int_{0}^{\pi }\exp\left(-q^{2}t_{d}D_{}\cos^{2}\vartheta \right)\sin\vartheta \;d\vartheta$$

Here, ϑ denotes the angle between pore axis and field gradient and the integral considers the powder average over the random orientations of the silica particles in our samples. For the analysis of the SFG data, we again took into account that the STE intensity also decayed owing to spin relaxation. Hence, it was necessary to supplement Equations (10) and (11) by appropriate damping functions, which could be determined, e.g., by identical measurements in homogeneous magnetic fields $B_{0}$, see below.

**Figure 9:**
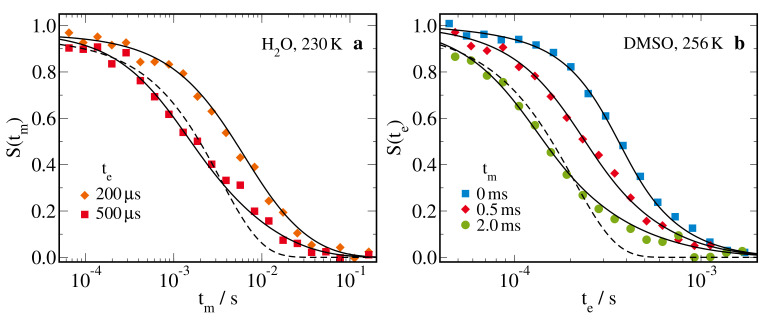
^1^H SFG STE decays of aqueous DMSO solutions in silica pores with a diameter of d=2.8 nm: (**a**) $S(t_{m})$ of 66 mol% H_2_O-DMSO-$d_{6}$ for the indicated evolution times $t_{e}$ at 230 K and (**b**) $S(t_{e})$ of 66 mol% D_2_O-DMSO-$h_{6}$ for various mixing times $t_{m}$ at 256 K, where data obtained from SFG Hahn-echo experiments are denoted as $t_{m}=0$. The solid lines indicate the results of global fits using the model of one-dimensional diffusion, see Equation (11). The dashed lines show fit results for the model of free diffusion, see Equation (10). They are included to indicate that the free-diffusion model fails to describe the NMR diffusion data for the silica pores.

Figure 9 displays ${}^{1}$H static field gradient (SFG) stimulated echo (STE) decays for aqueous DMSO solutions in silica pores with a diameter of d=2.8 nm. Specifically, results for H_2_O-DMSO-$d_{6}$, probing H${}_{2}$O diffusion, and D_2_O-DMSO-$h_{6}$, probing DMSO diffusion, are presented in panels (a) and (b), respectively. We see that the STE decays of both components showed a strong nonexponentiality and, hence, the model of free diffusion did not apply, as expected. Rather, the stretched shape was well described by the model of one-dimensional diffusion. Explicitly, this model supplemented by spin-relaxation damping enabled successful global interpolations of $S(t_{m},t_{e})$ data using a single value of the diffusion coefficient *D*, see Equation (11). This was illustrated for H${}_{2}$O diffusion based on $S(t_{m})$ decays for various evolution times $t_{e}$ and for DMSO diffusion considering $S(t_{e})$ data for various mixing times $t_{m}$. These findings imply that both solvent components were indeed confined to the silica pores and that their diffusion inside these confinements was observed. The associated diffusivities *D* are presented below.

${}^{1}$H NMR diffusion measurements in soft confinements require more sophisticated analysis [105,106,107]. In these studies, it is essential to consider that the observed signals receive contributions from proton species, which differ with respect to their self-diffusion and spin-relaxation behaviors. Moreover, it is necessary to take into account that the proton species can exchange magnetization via the chemical exchange of protons or a cross spin relaxation driven by residual dipolar couplings [108,109]. On the other hand, the matrix molecules mean restrictions to the diffusion process on length scales similar to their sizes, leading to obstructions for the transport process, but their disordered arrangement does not cause deviations from the model of free diffusion on the length scales of NMR diffusometry, i.e., ∼1 μm, so that Equation (10) rather than Equation (11) should be applicable.

**Figure 10:**
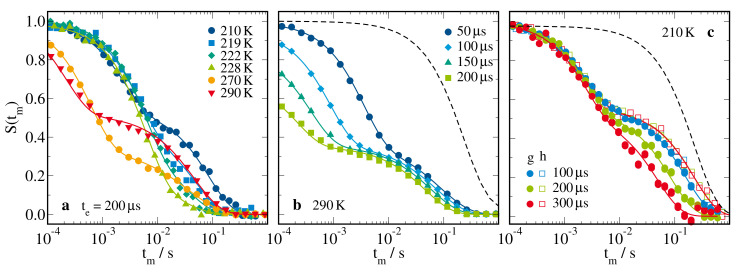
${}^{1}$H SFG STE decays of 66 mol% H_2_O-DMSO-$d_{6}$ in ficoll confinement at the solvent fraction $w_{s}=70\;$wt%: (**a**) $S(t_{m})$ for $t_{e}=200\;\mu$s at the indicated temperatures together with $S(t_{m})$ for various evolution times $t_{e}$ at (**b**) 290 K and (**c**) 210 K. In the latter panel, we compare results from identical measurements in gradient (g) and homogeneous (h) magnetic fields. The solid lines are fits to a superposition of the respective free-diffusion decays of two components at higher temperatures, see Equation (10), and to the free-diffusion decay of a faster component in the presence of cross relaxation to a slower component below 230 K, see Equation (12).

In Figure 10, we show ${}^{1}$H SFG STE decays of the H_2_O-DMSO-$d_{6}$ solution in ficoll confinement at temperatures 210–290 K. While the temperature dependence is shown in panel (a), the evolution-time dependence is presented for the highest and lowest temperature in panels (b) and (c), respectively. We see two-step decays at both high and low temperatures but the origin of the bimodality was completely different. At high temperatures, the two steps could be attributed to water and ficoll protons, consistent with the assignment in a diffusion study on neat water in ficoll confinement [110]. The short-time step strongly shifted to shorter times when $t_{e}$ and, thus, *q* increased indicative of fast water diffusion, while the long-time step showed a weaker evolution-time dependence, suggesting that slow ficoll diffusion interfered with additional spin-relaxation damping. Therefore, we fitted the SFG STE data at high temperatures to a superposition of two free-diffusion decays, see Equation (10). At low temperatures, the two-step decays had a more sophisticated interpretation, which was worked out in previous NMR approaches to diffusion in macromolecular matrices [105,106,107]. In detail, we need to consider that, as a result of the different molecular mobilities, the ficoll protons had much shorter spin–spin relaxation times $T_{2}$ than the water protons so that the signal of the former, unlike that of the latter, was eliminated during both evolution times $t_{e}$ of the experiment. Thus, only protons that belonged to the water subensemble both before and after the mixing time $t_{m}$ could contribute to the STE signals. Therefore, when magnetization was transferred between the water and ficoll protons by either chemical exchange or cross relaxation, the STE intensity decayed to a finite plateau, which indicated that a statistical distribution was reached. This STE decay was expected to be independent of the length of the evolution time, provided $t_{e}\gg T_{2}$ is valid, and of the strength of the field gradient *g*. In Figure 10c, we observe that the short-time decay of $S(t_{m})$ at 210 K met these criteria, and hence it did not result from molecular diffusion. In particular, it occurred not only in the gradient field but also in the homogeneous field, where diffusion was not probed. By contrast, the long-time decay of $S(t_{m})$ at 210 K depended on these experimental parameters. In the homogeneous field, the plateau at intermediate times, which was determined by the faction of water protons, finally decayed due to spin relaxation, independent of the value of $t_{e}$. By contrast, in the gradient field, the long-time decay shifted to shorter times when the evolution time was extended, indicating that water diffusion was observed. The magnetization exchange between different proton species can be taken into account by fitting the SFG STE decays at low temperatures to the model of Peschier et al. [105]

(12) $$S_{(}t_{m},t_{e})\propto \exp\left(-q^{2}t_{d}D\right)\left[\frac{a^{+}-k_{f}-R_{1f}}{a^{+}-a^{-}}\exp\left(-a^{+}t_{m}\right)-\frac{a^{-}-k_{f}-R_{1f}}{a^{+}-a^{-}}\exp\left(-a^{-}t_{m}\right)\right]$$

with

(13) $$a^{\pm }=\frac{1}{2}\left[q^{2}D+k_{w}+R_{1,w}+k_{f}+R_{1f}\pm \sqrt{{\left(q^{2}D+k_{w}+R_{1w}-k_{f}-R_{1f}\right)}^{2}+4k_{w}k_{f}}\right].$$

Here, $R_{1f}=1/T_{1f}$ and $R_{1w}=1/T_{1w}$ are the ${}^{1}$H SLR rates of the ficoll and the water protons, respectively, and $k_{f}$ ($k_{w}$) is the exchange rate from the ficoll (water) pool to the water (ficoll) pool. These parameters can be obtained from interpolating the STE decays in the homogeneous magnetic field with Equation (12). Keeping these parameters fixed, the self-diffusion coefficients *D* could readily be determined from the STE decays in the gradient field. In particular, we observe in Figure 10c that this fit approach well described their evolution-time dependence. Altogether, the water diffusivities were available from the short-time and long-time decays at high and low temperatures, respectively, while the onset of magnetization exchange led to intermingled contributions of the proton species and, hence, interfered with reliable analysis in the temperature range 230–270 K.

**Figure 11:**
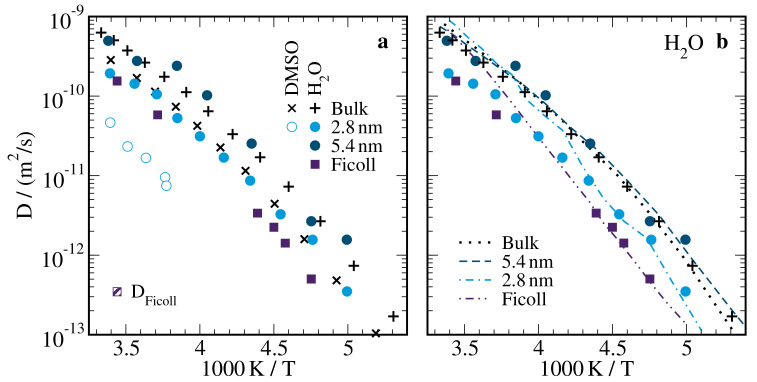
(**a**) Temperature-dependent self-diffusion coefficients *D* of the components of 66 mol% H_2_O-DMSO-$d_{6}$ (H${}_{2}$O) and D_2_O-DMSO-$h_{6}$ (DMSO) solutions in the bulk, in silica pores with the indicated diameters, and in ficoll at a mass fraction of $w_{s}=70$ wt%. The shaded square is the self-diffusion coefficient of ficoll. (**b**) Comparison of (symbols) the measured self-diffusion coefficients of H${}_{2}$O with (lines) *D* values calculated from the SLR correlation times τ of D${}_{2}$O, see Figure 8, based on the Stokes–Einstein–Debye relation Equation (14).

Figure 11a presents the self-diffusion coefficients *D* obtained from the ^1^H SFG STE studies on aqueous DMSO solutions in the bulk and in silica and ficoll confinements. Neglecting minor differences in the dynamics of H_2_O-DMSO-$d_{6}$ and D_2_O-DMSO-$h_{6}$ solutions, we found that, in the bulk, the diffusion coefficients of H${}_{2}$O were a factor of ∼3 larger than that of DMSO, essentially independent of temperature. This result is in harmony with the outcome of previous studies [47,49,51]. The common temperature dependence indicated a coupling of H${}_{2}$O/D${}_{2}$O and DMSO bulk dynamics, at least above 200 K. In silica confinement, the diffusion coefficients of H${}_{2}$O and DMSO depended on the pore diameter. Compared to the bulk behavior, the diffusivities were hardly altered in the wider pores with d=5.4nm, while they were significantly reduced in the narrower ones with d=2.8nm. These findings for the pore-size dependence of the self-diffusion coefficients are consistent with the above discussed variation of the rotational correlation times. In ficoll confinement, an even stronger slowdown of diffusion was observed. However, it cannot be excluded that exchange effects were not fully removed by the performed analysis and, hence, it is possible that the *D* values obtained for these samples did not describe the true diffusion coefficients of the components but some kind of average. Comparing the confinement effects for the components of the solution, we found that the diffusion was more retarded for the DMSO than the H${}_{2}$O molecules in the narrow silica pores, and hence the difference in their mobilities was further enhanced with respect to the bulk behavior.

Finally, we compare our NMR findings for the rotational and diffusive motions. The Stokes–Einstein–Debye (SED) relation predicts a coupling of both modes of motion according to

(14) $$D\tau_{2}=\frac{2}{9}R_{H}^{2}\;.$$

Here, $R_{H}$ is the hydrodynamic radius and $\tau_{2}$ is the correlation time associated with $F_{2}$. To test the validity of the SED relation for the water molecules in the studied samples, we used this approach to calculate diffusion coefficients *D* from the correlation times $\tau \equiv \tau_{2}$ obtained in our ${}^{2}$H SLR studies for the D${}_{2}$O reorientation. In doing so, the hydrodynamic radius was chosen for best overlap of measured and calculated diffusivities, resulting in $R_{H}=2.5\;$Å for the bulk solution, $R_{H}=2.2\;$Å for the silica pores, and $R_{H}=1.9\;$Å for the ficoll sample. In Figure 11b, it can be seen for the bulk solution that the diffusion coefficients measured in our SFG studies agreed with those calculated based on the SLR data down to ∼185 K, indicating that the SED relation was valid in a broad temperature range. While the same was true for the wider silica pores with d=5.4 nm, we found that the measured *D* values had a weaker temperature dependence than the calculated ones for the narrower silica pores with d=2.8 nm and the ficoll matrix. Analogous NMR studies of neat hydrogen-bonded liquids in the same silica confinements reported that the SED relation was obeyed for confined ethylene glycol [88], while the deviations from the SED relation were even stronger for confined water, independent of the pore size [36]. We emphasize that the obtained hydrodynamic radii $R_{H}$ should be taken with great care because D${}_{2}$O and H${}_{2}$O were used in the reorientation and diffusion studies, respectively. Moreover, the SED approach does not consider dynamical heterogeneity and neglects that diffusion coefficients and correlation times reflect diverse averages over mobility distributions, which can be very different. Thus, we expect that the prominent dynamical heterogeneity was the reason for the smaller hydrodynamic radii obtained for H${}_{2}$O in the confined solutions. The lack of correlation times $\tau_{2}$ for DMSO hampered analogous SED analyses for these molecules.

## 3. Conclusions

Combining BDS and NMR approaches, we ascertained rotational and diffusive molecular dynamics involved in the glassy slowdown of aqueous DMSO solutions in the bulk and in various confinements. For the most part, we focused on the eutectic composition, ensuring good glass-forming ability of the solutions. Furthermore, we used silica, ficoll, and lysozyme matrices to determine the dependence of the solution dynamics on the properties of the confinement. We found that both the bulk and the confined solutions show the general characteristics of glass-forming liquids. However, on a quantitative level, the dynamical scenario depends on the properties of the confinement.

In accordance with the bulk behavior, our BDS and NMR studies revealed that the α process of the confined aqueous DMSO solutions is characterized by a non-Arrhenius temperature dependence and a non-exponential time dependence. However, it is, in general, moderately slower and more heterogeneous than its bulk counterpart, depending on the nature of the confinement. In silica pores, we found that the rotational correlation times τ increase and the self-diffusion coefficients *D* decrease when the pore diameter is reduced to d=2.8 nm. Likewise, in ficoll matrices, the α process is more retarded for smaller weight fractions $w_{s}$ of the solution and, hence, smaller distances between the confining macromolecules. Furthermore the solution dynamics is more heterogeneous in narrower confinements, in particular, in ficoll and lysozyme ones, as indicated by broadened BDS processes I(a/b) and higher $T_{1}$ minima.

More detailed analyses showed that confinement affects various dynamical modes differently. In NMR diffusion studies, we exploited the isotope selectivity of the method to determine the diffusivities for both components of the solutions separately. We found that silica confinements cause a stronger decrease for the self-diffusion coefficients of the DMSO molecules than for those of the H${}_{2}$O molecules and, hence, they enhance the difference in the mobility of the components present in the bulk solution. We propose that this species-dependent slowdown arises because DMSO interacts more strongly with the silica walls than H${}_{2}$O and/or the fraction of molecules interacting with the walls is higher when the size ratio between the confinement and the molecule is smaller and, hence, for the bigger component more so than for the smaller one. Moreover, we observed that, unlike the bulk solution, the confined solutions do not obey the SED relation when the confinement is sufficiently severe, i.e., the temperature dependence of their reorientation and diffusion dynamics is different. In previous studies, such SED breakdown was also observed for confined water [36], whereas it did not occur for confined ethylene glycol [88].

Most of our findings for the α process of the confined aqueous DMSO solutions can be rationalized using core-shell models. Explicitly, the fraction of fast molecules in the core regions relative to that of the slow molecules in the shell regions is smaller in narrower confinements, resulting in a slowdown of the average dynamical behavior. Moreover, these mobility gradients across the confinement lead to more pronounced dynamical heterogeneity. In particular, bulk-like dynamics in the center results in bulk-like high-frequency flanks of dynamic susceptibilities, while slowed-down dynamics at the interface leads to strongly broadened low-frequency flanks, in harmony with findings in spatially-resolved simulation studies [30,31,32]. Finally, the observation that both effects are particularly prominent in the soft confinements can be traced back to the fact that the solution is confined to thin layers between the ficoll or lysozyme molecules for the studied compositions and, hence, there are small core-to-shell ratios and, probably, high mobility gradients. A detailed comparison of the actions of the ficoll and lysozyme confinements is, however, hampered by their irregular shapes and an influence of chemical exchange on the experimental results for the former systems.

Consistent with previous results for the bulk solution [57], we observed that a β process splits off from the α process near the glass transition temperature $T_{g}$. The properties of this β process are similar in all studied confinements and in the bulk solution. Moreover, they resemble those of the universal ν process in various kinds of aqueous systems. Based on these similarities, we conclude that the β process can be identified with the ν process. Consistently, our ${}^{2}$H STE studies indicated that it involves D${}_{2}$O reorientation. Accepting this assignment, the observed absence of confinement effects implies that the ν process is a more local relaxation than the α process.

## 4. Materials & Methods

### 4.1. Sample Preparation

The preparation and characterization of the mesoporous silica materials was done in previous works [36,87]. Briefly, MCM-41 with a pore diameter of d=2.8 nm was synthesized by the Buntkowsky group (Chemistry Department, TU Darmstadt) and SBA-15 with a pore diameter of d=5.4nm was purchased from Sigma-Aldrich. Pore volumes and diameters were determined by nitrogen gas adsorption. Scanning electron microscopy showed that the MCM-41 particles had nearly spherical shapes and particle sizes of ∼400 nm [36,85,87]. Lysozyme from chicken egg white, ficoll (Ficoll PM 70, M=70kDa), H${}_{2}$O, D${}_{2}$O, DMSO-$d_{6}$, and DMSO-$h_{6}$ were purchased from Sigma-Aldrich. All chemicals were used as received. However, spurious amounts of water were removed from the silica and lysozyme matrices in high vacuum prior to use [77].

The sample compositions were assured by weighing the components. Mesoporous silica were completely loaded with the aqueous DMSO solutions. In doing so, the knowledge of the specific pore volume was used to determine the amount of solution required for ∼100 % filling. The ficoll- and lysozyme-based materials were prepared by carefully mixing the components. The solvation level of lysozyme was chosen to correspond to a fraction of 1.6 solvent molecules per amino acid, independent of the composition of the solution. This criterion resulted in solvation levels of $s=0.68\;g_{\mathrm{sol}}/g_{\mathrm{lys}}$ and $s=0.97\;g_{\mathrm{sol}}/g_{\mathrm{lys}}$ for the studied D${}_{2}$O-DMSO-$h_{6}$ solutions with water mole fractions of 66 and 33 mol%, respectively. For all samples, we observed shelf-times of several days to assure equilibration prior to the measurements. All NMR samples were sealed in the NMR tubes to avoid solvent losses during the long-lasting NMR measurements.

### 4.2. Broadband Dielectric Spectroscopy and Nuclear Magnetic Resonance Experiments

The complex permittivity $\varepsilon^{*}\left(\nu \right)$ was recorded in the frequency range from $10^{-2}\;$Hz to $10^{6}\;$Hz using a Novocontrol Alpha-N analyzer. The sample temperature was controlled by a Novocontrol Quatro Cryosystem with a temperature stability better than ±0.1K and an accuracy of ±0.5K.

The ${}^{2}$H NMR experiments were performed using home-built spectrometers operating at a ${}^{2}$H Larmor frequency of $\omega_{0}=2\pi \cdot 46.1\;$MHz. SLR measurements used a saturation-recovery sequence with solid-echo detection. For STE measurements, we employed a four-pulse sequence, which allows one to overcome the receiver dead time, and an appropriate phase cycle to eliminate undesired coherences [111]. Further details about the ${}^{2}$H NMR setups can be found in previous studies [57,90].

The ${}^{1}$H NMR diffusion measurements utilized a specially designed SFG setup [112]. The measurements were carried out at two sample positions where the Larmor frequencies amount to $\omega_{0}=2\pi \cdot 165$ MHz and $\omega_{0}=2\pi \cdot 92$ MHz and the field gradients to g=140 T/m and g=110 T/m, respectively. To determine relaxation contributions to the ${}^{1}$H SFG NMR data, we performed identical experiments in a homogeneous magnetic field at the Larmor frequency $\omega_{0}=2\pi \cdot 92$ MHz. Further details about the NMR diffusion measurements can be found in previous works [36,107].

## Supplementary Materials

Additional dielectric spectra are available online at.

## Abbreviations

The following abbreviations are used in this manuscript:

BDS	Broadband dielectric spectroscopy
CC	Cole–Cole
DMSO	Dimethyl sulfoxide
MWS	Maxwell–Wagner–Sillars
NMR	Nuclear magnetic resonance
SLR	Spin-lattice relaxation
SED	Stokes–Einstein–Debye
SEI	Solid-echo intensity
STE	Stimulated echo
SFG	Static field gradient
VFT	Vogel–Fulcher–Tammann
