# Supplementary material for: Confinement Effects on Glass-Forming Aqueous Dimethyl Sulfoxide Solutions
Source: Molecules. 2020 Sep 9;25(18):4127. doi: 10.3390/molecules25184127 (PMC7570821; doi:10.3390/molecules25184127)
Supplement: Supplementary file 1 [file molecules-25-04127-s001.pdf]

Supplementary Materials: Confinement effects on glass-forming aqueous dimethyl sulfoxide solutions

Dominik Demuth, Melanie Reuhl, Moritz Hopfenmüller, Nail Karabas, Simon Schoner, and Michael Vogel

Figure S1 shows temperature-dependent dielectric loss spectra $\varepsilon''(\nu)$ of D_2O-DMSO-h_6 in lysozyme for two compositions of the solution, explicitly, for water mole fractions of $c_W = 66\,\%$ and $c_W = 33\,\%$. Figure S2 displays temperature-dependent dielectric loss spectra $\varepsilon''(\nu)$ of D_2O-DMSO-h_6 solution in ficoll at mass fractions of $w_s = 70\,\%$ and $w_s = 40\,\%$.

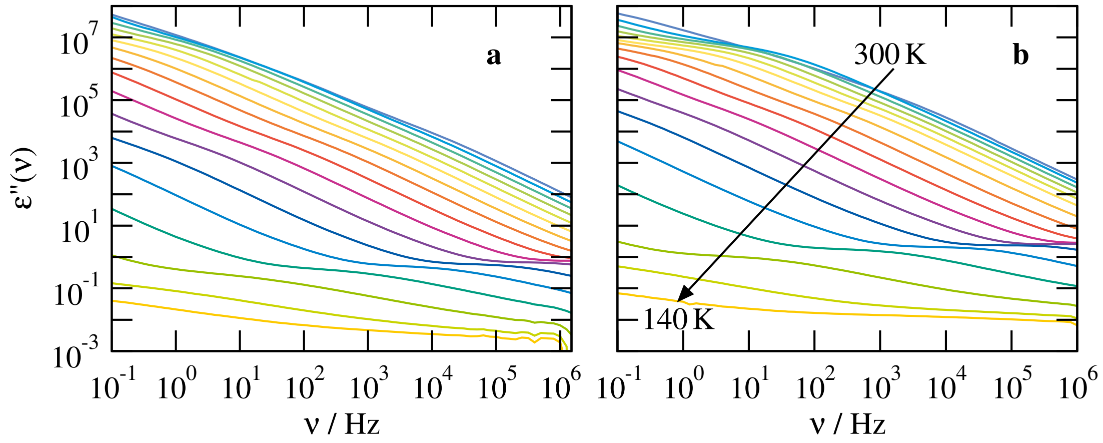

Figure S1. Dielectric loss spectra $\varepsilon''(\nu)$ of D_2O-DMSO-h_6 in lysozyme for two compositions of the solution in the temperature range 140–300 K: (**a**) D_2O:DMSO ratio of 2:1 and (**b**) D_2O:DMSO ratio of 1:2.

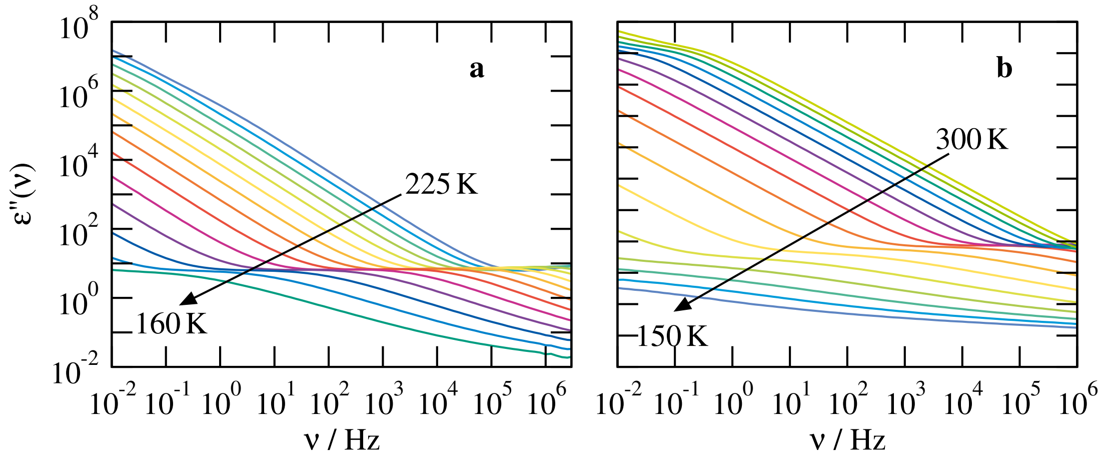

Figure S2. Dielectric loss spectra $\varepsilon''(\nu)$ of 66 mol% D_2O-DMSO-h_6 solution in ficoll at mass fractions of (**a**) $w_s = 70\,\%$ and (**b**) $w_s = 40\,\%$. The covered temperature ranges are indicated.
